# Eliminating Cervical Cancer: the Impact of Screening and Human Papilloma Virus Vaccination

**DOI:** 10.5888/pcd22.250127

**Published:** 2025-07-24

**Authors:** Samah Nabi, Brenda-Ruth Mimba, Ogochukwu Akunne

**Affiliations:** 1 Morehouse School of Medicine. Atlanta, Georgia

Cervical cancer is a vaccine-preventable disease that is a significant public health concern because of its considerable impact on disease and death among women (1). The National Cancer Institute estimated that as of 2021 approximately 295,000 women in the US had been diagnosed with this cancer, with 7.6 per 100,000 women newly diagnosed per year (2). Cervical cancer is unique among cancers because of its well-defined pathogenesis (3). Approximately 99.7% of these cancers are due to untreated or chronic infection with human papillomavirus (HPV), a sexually transmitted virus that directly infects the squamous cell epithelium on mucosal surfaces (3). The 5-year relative survival rate of cervical cancer is 67.4%, but when diagnosed at an early stage, the survival rate rises significantly, to 91% (2). Early vaccination against HPV has proven successful in preventing this cancer, with early vaccination resulting in a 40% reduction in cervical precancers and more than an 80% reduction in the overall risk of developing the disease (4,5). Prevention and early detection are critical to improving survival (6).

The elimination of cervical cancer is primarily dependent on increasing HPV vaccination rates, implementing cervical cancer screening programs, and increasing education. However, several barriers stand in the way, including health care access, insurance status, vaccine hesitancy, limited public education, stigma, and cost.

## HPV Vaccination

Unlike many other cancers, HPV infections and the risk of cervical cancer can be eliminated (5). As of 2019, the US Food and Drug Administration had approved 3 HPV vaccines: Gardasil-9 (9-valent/9vHPV), Gardasil (quadrivalent/4vHPV), and Cervarix (bivalent/2vHPV), to prevent HPV infections (7). While all 3 vaccines are effective against HPV types 16 and 18, the most prevalent strains in cancer development, the US administers only Gardasil-9 because of its broad-spectrum protection against multiple HPV types (7–9). Still, all 3 vaccines have been proven highly effective in preventing cervical cancer by preventing infection with HPV (10).

The Centers for Disease Control and Prevention (CDC) recommends that people receive an HPV vaccination between ages 9 through 26 years, with earlier vaccination preferred as it prevents future HPV infections (5). As of 2022, the overall vaccination rate (at least 1 HPV dose) for US adolescents aged 13 to 17 years was 76% (11). However, rates vary among states, with several reporting that less than 70% of adolescents have had at least 1 HPV vaccine and less than 55% were up to date with HPV vaccination (11).

## Barriers to HPV Vaccination and Cervical Cancer Screening

Given the existence of an established vaccine for preventing HPV infection and thereby reducing cervical cancer risk, incidence of that cancer should be significantly reduced. However, barriers such as limited health care access, insurance status, vaccine hesitancy, limited public education, stigma, and cost diminish the impact of the vaccine (12–14). 

Cost and lack of insurance coverage have been consistently cited as major obstacles to HPV vaccination (15). Although increased vaccination rates have been linked with increased screening, studies show that only 68% of people in the US are aware of HPV, its relationship to certain cancers, and the availability of vaccines to prevent infection (16,17). This knowledge gap is greater among African American and Hispanic populations than among their White counterparts, potentially contributing to the racial disparities observed in vaccination rates (7). A 2021 study found that although 48% of White women received the HPV vaccine, only 38% of African American women and 30% of Hispanic women received it (17). A large proportion of Hispanic women reported not having undergone cervical cancer screening because they did not know that they needed it (18). Furthermore, women without health insurance reported 7 times more often that they could not receive screening because of lack of access compared with women with private health insurance (18). Many of these barriers were further compounded by the COVID-19 pandemic, which saw a decrease in HPV vaccine delivery and administration because of the shift to pandemic-related health priorities (19,20). The pandemic also affected the ability of children and adults to schedule routine visits, and a higher proportion of those who were unable to schedule visits were from racial and ethnic minority groups or were living below the poverty level (21). Also, vaccine hesitancy and refusal increased during the COVID-19 pandemic, which further limited the impact of the HPV vaccination (19,22). These disparities highlight the need for programs that provide information to minority and socioeconomically marginalized populations, alleviate the structural barriers that prevent patients from obtaining screening and vaccination, and ensure adherence to national vaccination and screening guidelines.

## Preventive Measures for Reducing Cervical Cancer

While HPV vaccination plays a critical role in reducing the risk of cervical cancer, several other preventive measures are important. The World Health Organization (WHO) has established the Cervical Cancer Elimination Initiative, which outlines a plan for all countries to maintain an incidence rate less than 4 women per 100,000 by 2030 (23). The plan is based on a 90–70–90 target: 90% of girls fully vaccinated with the HPV vaccine by the age of 15, 70% of women screened with a high-performance test by the age of 35 years and again by the age of 45, and 90% of women with precancer treated and 90% with invasive cancer managed (23). WHO supports many primary prevention measures, such as sexual and reproductive health education, education about protective sex practices, and programs that promote healthy lifestyles in children and adolescents (23). The success of these measures hinges on understanding social, cultural, and societal barriers that can affect the success of HPV vaccination (23). WHO also supports secondary prevention measures related to the reduction of cervical cancer incidence and mortality, the identification and treatment of precancerous lesions (23).

The Papanicolaou (Pap) test, a cervical cytology test, samples and analyzes cells from the vagina and cervix to detect abnormalities (10). This test has become the standard in cervical cancer screening in developed countries and has led to a 70% decrease in incidence and mortality from this cancer (10). For high-risk women aged 30 years or older, WHO recommends HPV–DNA testing along with a Pap test (10). Application of these guidelines is most successful in areas with established health care infrastructure where patients can receive follow-up care. In areas with low health care resources and among rural populations, the benefit of routine Pap tests is limited due to low sensitivity and specificity (23). Instead, self-sampling, rapid HPV testing, and visual inspection of the cervix with acetic acid have been more successful, which is likely due to availability of HPV rapid testing self-test kits (23). If precancerous lesions are identified, treatment depends on their severity and can include various ablation methods or surgical excision (10).

## Initiatives to Increase HPV Vaccination and Cervical Cancer Screening

Several countries have established programs in response to WHO’s initiatives to help increase cervical cancer screening and HPV vaccination rates. For example, Australia has implemented an aggressive HPV vaccination program for children and adolescents that achieved an 80% full vaccination rate among girls aged 15 years or younger (24). In addition, 67% of women aged 45 to 49 years were screened, and 86% of precancerous lesions were treated under this program within 6 months of diagnosis (24). In India, mobile health education interventions among women from low socioeconomic backgrounds achieved a significant increase in knowledge about cervical cancer and HPV vaccination and a 5% increase in screening after the educational intervention (25).

In the US, initiatives such as Alabama’s Operation Wipeout (26) and CDC’s National Breast and Cervical Cancer Early Detection Program (NBCCEDP) (27) are providing HPV vaccination and cervical cancer screening to women in low-income regions of the US. Operation Wipeout is a partnership among Alabama’s public, private, academic, and nonprofit sectors started by researchers at the University of Alabama at Birmingham. The initiative established goals that model the WHO’s 90–70–90 targets and aims to increase the initiation of HPV vaccination (receipt of a single dose) to 90% among children aged 9 to 12 years and 85% among adolescents aged 13 to 17 years, to increase HPV vaccination dose completion (receipt of 3 doses) to 80% in both age groups, to increase compliance with screening guidelines to 90%, and to increase adherence to cervical cancer treatment and follow-up to 90% (26). NBCCEDP is a federally funded program that provides Pap tests, HPV tests, and pelvic examinations to women who are uninsured, underinsured, or living below the poverty line (27). In 2023, the NBCCEDP provided cancer screening and diagnostic services to approximately 129,000 women in the US (27). As a result of that program, 87 invasive cervical cancers and approximately 6,200 precancerous cervical lesions were detected (28).

While these initiatives show promise, outcomes still fall short of the targets set by WHO. Social, cultural, and societal norms affected these initiatives in cervical cancer. Black, Latina, and Chinese American women with cervical cancer reported feeling self-blame and experiencing both internalized and public stigmas related to their diagnosis, leading to isolating behaviors and negative health outcomes (29). Additionally, fear, embarrassment, and anxiety are often cited as major barriers to adherence to screening guidelines (15). Addressing these psychological barriers, in addition to socioeconomic challenges that prevent women from getting adequate care, is essential to improving screening rates and outcomes for women with cervical cancer.

## Conclusion

The US has the resources necessary to provide widespread access to HPV vaccination and cervical cancer screening. However, disparities in health care access persist, especially among women in racial and ethnic minority communities and rural areas and among women of low socioeconomic status. Initiatives like NBCCEDP and Operation Wipeout can help address these disparities by providing free or low-cost screening and HPV vaccinations and by providing educational resources tailored to medically underserved communities (26,27). Culturally relevant educational materials can help reduce the stigma associated with cervical cancer screening. Telehealth can enhance health care accessibility by distributing educational resources and improving health care access among women in low-income and rural areas. Most importantly, initiatives, both domestically and internationally, have shown the impact of proper funding and management in increasing rates of HPV vaccination and screening. For the long-term success of these strategies, the US must continue to fund programs and advance policies that emphasize minority and disadvantaged populations to reach the goals established by WHO and eliminate cervical cancer.
